# Supplementary material for: Multi‐dataset identification of innovative feature genes and molecular mechanisms in keratoconus
Source: J Cell Mol Med. 2024 Sep 19;28(18):e70079. doi: 10.1111/jcmm.70079 (PMC11412914; doi:10.1111/jcmm.70079)
Supplement: Supplementary file 2 — Material S2. [file JCMM-28-e70079-s002.zip › 7.GSEA analysis/3.DBNDD1/KEGG_OOCYTE_MEIOSIS.html]

Details for gene set KEGG\_OOCYTE\_MEIOSIS[GSEA]

|  || Dataset | DBNDD1.group.cls#h\_versus\_l |
| Phenotype | group.cls#h\_versus\_l |
| Upregulated in class | h |
| GeneSet | KEGG\_OOCYTE\_MEIOSIS |
| Enrichment Score (ES) | 0.14029269 |
| Normalized Enrichment Score (NES) | 0.70119226 |
| Nominal p-value | 0.8989899 |
| FDR q-value | 1.0 |
| FWER p-Value | 1.0 |
Table: GSEA Results Summary

  

Fig 1: Enrichment plot: KEGG\_OOCYTE\_MEIOSIS      
 Profile of the Running ES Score & Positions of GeneSet Members on the Rank Ordered List

  

| SYMBOL | TITLE | RANK IN GENE LIST | RANK METRIC SCORE | RUNNING ES | CORE ENRICHMENT || 1 | CAMK2B | na | 217 | 0.536 | 0.0553 | Yes |
| 2 | PPP3R2 | na | 1210 | 0.361 | 0.0681 | Yes |
| 3 | CAMK2A | na | 3995 | 0.213 | 0.0122 | Yes |
| 4 | CALML6 | na | 4335 | 0.202 | 0.0256 | Yes |
| 5 | CPEB1 | na | 4808 | 0.186 | 0.0333 | Yes |
| 6 | CCNE2 | na | 4812 | 0.186 | 0.0546 | Yes |
| 7 | ADCY7 | na | 6033 | 0.150 | 0.0365 | Yes |
| 8 | AR | na | 6232 | 0.144 | 0.0474 | Yes |
| 9 | INS | na | 6241 | 0.144 | 0.0637 | Yes |
| 10 | ADCY9 | na | 6468 | 0.137 | 0.0730 | Yes |
| 11 | ADCY6 | na | 6632 | 0.133 | 0.0836 | Yes |
| 12 | ANAPC1 | na | 6638 | 0.133 | 0.0987 | Yes |
| 13 | RPS6KA2 | na | 7249 | 0.119 | 0.0947 | Yes |
| 14 | FBXO43 | na | 7799 | 0.107 | 0.0912 | Yes |
| 15 | CALML5 | na | 7930 | 0.105 | 0.0995 | Yes |
| 16 | IGF1R | na | 8164 | 0.100 | 0.1043 | Yes |
| 17 | ADCY4 | na | 8335 | 0.097 | 0.1106 | Yes |
| 18 | ADCY5 | na | 8470 | 0.095 | 0.1177 | Yes |
| 19 | ESPL1 | na | 8518 | 0.094 | 0.1272 | Yes |
| 20 | CHP2 | na | 8667 | 0.091 | 0.1334 | Yes |
| 21 | PPP2R1B | na | 8784 | 0.089 | 0.1403 | Yes |
| 22 | MAPK3 | na | 9865 | 0.073 | 0.1175 | No |
| 23 | CALML3 | na | 10231 | 0.068 | 0.1148 | No |
| 24 | IGF1 | na | 10555 | 0.064 | 0.1128 | No |
| 25 | ITPR2 | na | 10876 | 0.060 | 0.1104 | No |
| 26 | CALM2 | na | 11915 | 0.048 | 0.0859 | No |
| 27 | SPDYC | na | 12239 | 0.045 | 0.0817 | No |
| 28 | PPP1CA | na | 12524 | 0.042 | 0.0784 | No |
| 29 | STAG3 | na | 12701 | 0.041 | 0.0780 | No |
| 30 | SMC1B | na | 13223 | 0.036 | 0.0671 | No |
| 31 | RBX1 | na | 13500 | 0.034 | 0.0631 | No |
| 32 | CAMK2G | na | 13529 | 0.034 | 0.0661 | No |
| 33 | FBXW11 | na | 13809 | 0.032 | 0.0617 | No |
| 34 | ANAPC4 | na | 14705 | 0.025 | 0.0387 | No |
| 35 | CDC23 | na | 14862 | 0.024 | 0.0369 | No |
| 36 | BTRC | na | 15199 | 0.021 | 0.0297 | No |
| 37 | PGR | na | 15265 | 0.021 | 0.0302 | No |
| 38 | PRKX | na | 15799 | 0.017 | 0.0168 | No |
| 39 | CALM1 | na | 15870 | 0.017 | 0.0167 | No |
| 40 | ANAPC11 | na | 16026 | 0.016 | 0.0140 | No |
| 41 | PPP2R5A | na | 16418 | 0.013 | 0.0042 | No |
| 42 | SLK | na | 16486 | 0.013 | 0.0038 | No |
| 43 | PPP2R5D | na | 16710 | 0.011 | -0.0014 | No |
| 44 | RPS6KA3 | na | 16816 | 0.011 | -0.0032 | No |
| 45 | YWHAB | na | 16820 | 0.011 | -0.0021 | No |
| 46 | ITPR3 | na | 16903 | 0.010 | -0.0033 | No |
| 47 | ANAPC5 | na | 17097 | 0.009 | -0.0079 | No |
| 48 | PPP2R5C | na | 17179 | 0.008 | -0.0093 | No |
| 49 | CCNE1 | na | 17317 | 0.007 | -0.0124 | No |
| 50 | ANAPC7 | na | 17646 | 0.005 | -0.0212 | No |
| 51 | CUL1 | na | 17786 | 0.004 | -0.0248 | No |
| 52 | PPP1CB | na | 17957 | 0.003 | -0.0293 | No |
| 53 | MOS | na | 18122 | 0.002 | -0.0338 | No |
| 54 | PPP3R1 | na | 18349 | 0.000 | -0.0403 | No |
| 55 | ANAPC13 | na | 18434 | -0.000 | -0.0427 | No |
| 56 | ADCY2 | na | 19167 | -0.006 | -0.0632 | No |
| 57 | SKP1 | na | 19198 | -0.006 | -0.0634 | No |
| 58 | ITPR1 | na | 19252 | -0.006 | -0.0642 | No |
| 59 | PPP2R1A | na | 19616 | -0.010 | -0.0735 | No |
| 60 | YWHAZ | na | 19676 | -0.010 | -0.0741 | No |
| 61 | ANAPC10 | na | 19847 | -0.012 | -0.0777 | No |
| 62 | PTTG2 | na | 19926 | -0.012 | -0.0785 | No |
| 63 | CDC16 | na | 20107 | -0.014 | -0.0821 | No |
| 64 | SMC1A | na | 20200 | -0.015 | -0.0830 | No |
| 65 | PTTG1 | na | 20284 | -0.015 | -0.0837 | No |
| 66 | CALM3 | na | 20684 | -0.019 | -0.0930 | No |
| 67 | PPP1CC | na | 20793 | -0.020 | -0.0938 | No |
| 68 | PRKACA | na | 20814 | -0.020 | -0.0921 | No |
| 69 | PPP2R5E | na | 21740 | -0.029 | -0.1154 | No |
| 70 | YWHAG | na | 22158 | -0.034 | -0.1236 | No |
| 71 | CDC26 | na | 22265 | -0.035 | -0.1227 | No |
| 72 | YWHAE | na | 22319 | -0.035 | -0.1201 | No |
| 73 | MAD2L1 | na | 22334 | -0.035 | -0.1165 | No |
| 74 | RPS6KA6 | na | 22401 | -0.036 | -0.1142 | No |
| 75 | CHP1 | na | 22407 | -0.036 | -0.1102 | No |
| 76 | PPP3CA | na | 22457 | -0.037 | -0.1074 | No |
| 77 | PPP2R5B | na | 22517 | -0.037 | -0.1048 | No |
| 78 | MAPK1 | na | 22705 | -0.040 | -0.1056 | No |
| 79 | ANAPC2 | na | 22849 | -0.041 | -0.1050 | No |
| 80 | PPP3CB | na | 22975 | -0.043 | -0.1037 | No |
| 81 | YWHAQ | na | 23046 | -0.044 | -0.1008 | No |
| 82 | ADCY8 | na | 23069 | -0.044 | -0.0963 | No |
| 83 | ADCY3 | na | 23170 | -0.045 | -0.0940 | No |
| 84 | CDC27 | na | 23312 | -0.047 | -0.0927 | No |
| 85 | PLCZ1 | na | 23423 | -0.048 | -0.0903 | No |
| 86 | PPP2CA | na | 23696 | -0.052 | -0.0922 | No |
| 87 | FBXO5 | na | 23879 | -0.054 | -0.0912 | No |
| 88 | PPP2CB | na | 24140 | -0.058 | -0.0920 | No |
| 89 | REC8 | na | 24247 | -0.060 | -0.0882 | No |
| 90 | SMC3 | na | 24731 | -0.067 | -0.0945 | No |
| 91 | MAD2L2 | na | 25657 | -0.081 | -0.1119 | No |
| 92 | MAPK12 | na | 25666 | -0.081 | -0.1027 | No |
| 93 | RPS6KA1 | na | 25675 | -0.081 | -0.0936 | No |
| 94 | CDK1 | na | 25994 | -0.087 | -0.0928 | No |
| 95 | CDC20 | na | 26050 | -0.089 | -0.0842 | No |
| 96 | PRKACB | na | 26730 | -0.102 | -0.0921 | No |
| 97 | PPP3CC | na | 26734 | -0.102 | -0.0804 | No |
| 98 | CCNB1 | na | 26847 | -0.104 | -0.0717 | No |
| 99 | SPDYA | na | 26999 | -0.108 | -0.0637 | No |
| 100 | PKMYT1 | na | 27115 | -0.110 | -0.0543 | No |
| 101 | CCNB2 | na | 27443 | -0.118 | -0.0503 | No |
| 102 | AURKA | na | 27454 | -0.118 | -0.0370 | No |
| 103 | PRKACG | na | 28125 | -0.134 | -0.0410 | No |
| 104 | CDK2 | na | 28335 | -0.139 | -0.0310 | No |
| 105 | MAP2K1 | na | 29246 | -0.164 | -0.0385 | No |
| 106 | YWHAH | na | 29598 | -0.176 | -0.0284 | No |
| 107 | ADCY1 | na | 29881 | -0.185 | -0.0153 | No |
| 108 | BUB1 | na | 31777 | -0.253 | -0.0410 | No |
| 109 | CDC25C | na | 32054 | -0.267 | -0.0184 | No |
| 110 | CAMK2D | na | 32964 | -0.326 | -0.0072 | No |
| 111 | PLK1 | na | 34377 | -0.506 | 0.0101 | No |
Table: GSEA details [plain text format]

  

Fig 2: KEGG\_OOCYTE\_MEIOSIS      
 Blue-Pink O' Gram in the Space of the Analyzed GeneSet

  

Fig 3: KEGG\_OOCYTE\_MEIOSIS: Random ES distribution      
 Gene set null distribution of ES for **KEGG\_OOCYTE\_MEIOSIS**
